# FPGA-Based Autonomous GPS-Disciplined Oscillatorsfor Wireless Sensor Network Nodes

**DOI:** 10.3390/s22093135

**Published:** 2022-04-20

**Authors:** Toan Quang The Bui, Arul Elango, René Jr. Landry

**Affiliations:** École de Technologie Supérieure, Université du Quebéc, Montréal, QC H3C 1K3, Canada; arul.elango@ens.etsmtl.ca (A.E.); rene.landry@etsmtl.ca (R.J.L.)

**Keywords:** TCXO, GPSDO, timing correction, Kalman filter, FPGA, wireless sensor network

## Abstract

Numerous devices in distributed wireless sensor arrays require a high-accuracy timing reference. Although the GPS-disciplined oscillators have been developed for decades, the hardware design still has performance limitations. In this context, we present the hardware implementation for a GPS-disciplined oscillator with an automatic adaptive drift correction algorithm, which is implemented in a low-cost, high-speed field-programmable gate array (FPGA) device. The system design and the hardware implementation are presented to demonstrate the advantages of the proposed oscillator. To verify this oscillator in real-time applications, we tested the device in multiple environments and compared it to state-of-the-art designs. The experimental results showed that our proposed device has a low cost and high performance. This device can achieve less than 80 ns and 356 ns in 1PPS signal drift in the indoor environment test and the outdoor environment test, respectively, after 24 h of working without a GPS signal.

## 1. Introduction

High-precision Global Positioning System (GPS)-disciplined oscillators (GPSDOs) have been designed to work as a source of reference timing, whose output is controlled to agree with the clock signals generated and broadcast by GPS in the Global Navigation Satellite System (GNSS) [1,2]. GPSDOs are widely used in measurement devices and communication systems due to their cost-effective, high-precision, and self-calibrating operation. The work in [3] presented the use of GPSDOs in synchronizing network radar. The GPS-disciplined clock was also used in an analog-to-digital converter (ADC) for phase measurement application as presented in [4]. Moreover, GPS signals have been used in wireless sensor networks (WSNs) for positioning the sensor nodes in recent research [5,6,7]. In this context, the timing calibration and synchronization between local oscillators in devices and the references are important to obtain high-accuracy results. The commonly used GPS modules can perform time synchronization with a resolution of 100 ns or smaller. Numerous GPSDOs are available on the market and in research laboratories. Four devices of one specific low-cost GPSDO type are characterized and compared using a tested GPSDO combining a GNSS signal simulator and a reference signal stability measurement system based on software-defined radio (SDR) and digital signal processing (DSP) [8].

However, there are several factors that would affect the accuracy of the frequency correction [9]. The first is the random errors and outlines contained in one pulse per second (PPS), which is the broadcast output of the GPSs [10]. The second factor is the frequency nonlinear drift of the crystal oscillators because of the aging and the temperature [11]. Therefore, several works have been developed to filter designs to eliminate the noise and adaptive algorithms are applied to compensate the temperature and aging effects [12,13]. However, most of the previous designs have a shortage of investigating the relationship between frequency variation and temperature change. In this paper, we present an adaptive algorithm based on Kalman filter models for temperature and aging effects. Moreover, a test is performed to verify the relationship between the temperature of the GPSDO and the frequency variation at the TCXO.

Additionally, as the GPS signals received are highly sensitive to the environment, especially in moving objects or in complex geography, the GPS-based timing systems occasionally lose their accuracy. Therefore, the main focus of this work is to design the GPS-disciplined oscillator (GPSDO), which achieves a high-precision self-calibration when the GPS signals from the satellites are not available. Many different factors influence the performance of the accuracy of receiving the 1PPS reference signal from the source and to maintain the accuracy of 1 μs during the non-availability of the GPS signal in disruptive conditions. Therefore, there is a need for a low-cost, low-power, compact-size, more robust, and re-configurable hardware architecture for developing the timing board that can be upgraded or tailored to various standards running on a high-speed, low-power processor to make it possible to compensate the temperature calibration and drift in the frequency within the allowable accuracy level.

In designing GPSDOs, the data acquisition (DAQ) is an important part that directly affects the performance of GPSDOs. Conventional GPSDOs require long time constants of integration to inherit the GPS signal’s long-term accuracy [14,15]. The higher the frequency of DAQ that is used, the smaller the time step achieved. Then, the accuracy of the system is increased while the latency is shortened. However, previous works developed the DAQ and the adaptive algorithms in embedded microprocessors, which have a limited operating frequency (a maximum of 20 MHz) [16,17]. Therefore, in this work, we concentrated on developing the integrated hardware architecture for utilizing the central processing unit of the Spartan-7 FPGA, which provides a 200 MHz operating frequency, integrated with the GNSS module, the voltage-controlled TCXO (VC-TCXO), and the temperature sensor for improving the timing error to less than 1 μs.

To verify the design’s performance, we provide multiple tests under different room temperatures in the indoor and outdoor environment to prove the capability of the autonomous embedded timing system in real applications. The results obtained with the newly designed timing board indicate that the improvement in the speed in clock counting is achieved by replacing the microcontroller with the FPGA chip, and the marginal stabilization time and frequency/temperature stability can also be attained by introducing a compact-size, low-cost alternative oscillator VC-TCXO.

From the testing results, it was also inferred that the estimated time errors are close to the specification requirement of the timing board. On very few occasions, the estimated timing error of 20% crossed the requirement of 1 μs. Further, to improve the results, we suggest continuing to design new Kalman filter algorithms on FPGAs to replace the old mean filter algorithms, which seem to regain the timing error within the bound running on this framework. In the new algorithm, we also plan to incorporate the aging and temperature parameter to improve the disciplined ability of this system.

The remainder of this paper is organized as follows. Related works are reviewed in Section 2. The system model of the proposed FPGA-based autonomous GPS-disciplined oscillator is presented in detail in Section 3. The hardware implementation and the tools are detailed in Section 4. The measurement method is mentioned and clarified in Section 5. The experimental results are presented and discussed in Section 6. Finally, Section 7 summarizes the work in this paper.

## 2. Related Works

To solve the previously addressed problems, the following state-of-the-art works are presented. The authors in [12] proposed a predictive finite impulse response (FIR) filter to synchronize the local clock using the GPS 1PPS signal. The work in [13] presented an adaptive unscented Kalman filter for GPSDOs, which provides high precision. However, the algorithm was simulated using MATLAB, while the implementation of the frequency difference measurement module was not clearly presented.

The work presented in [18] characterized four different GPSDOs under test in distributed vehicle-to-X (V2X) measurement applications. The 1PPS and 10 MHz signals were collected from four GPSDOs and compared in different modes (stationary and on-road tests, with/without the GPS reference signal). These devices accumulate a maximum of 133 μs of time error within 24 h, compared to the reference signal.

In [19], the authors provided the quality evaluation of the 1PPS signals in commercial devices, which can mitigate the systematic error of the reference clock. However, the practical design to obtain a high-accuracy timing clock was not presented. The authors in [20] developed the adaptive unscented Kalman filter to compensate for the temperature variation. Even though the model was simulated successfully in MATLAB, the experimental results were not provided. As the frequency difference is the input signal, its accuracy is a crucial factor that has a great impact on the output’s performance. The method and system design of a local reference clock by using the GPS 1PPS signal were proposed in [21].

Some works have been presented recently in hardware design to accomplish high-accuracy results for the generated 1PPS signal. As the frequency for the counter increases, so does the precision of the measurement. It should increase the counter frequency or extend the measurement window time. The work in [16] used a gate trigger to start and stop counting the 20MHz local oscillator. This hardware design was implemented in the complex programmable logic device (CPLD). However, due to the frequency-counting mechanism, the system had a long latency. as implemented in the complex programmable logic device (CPLD). However, due to the frequency-counting mechanism, the system had a long latency.

These previous works presented GPSDOs’ applications and improved the performance of generated clock with adaptive algorithms. However, the practical design of GPSDOs and the evaluation of their performance in real-time applications have not been addressed properly. Therefore, in this paper, we present our hardware design of an autonomous GPSDO in an FPGA, which provides a high frequency for data acquisition (DAQ). Then, the latency of the system is reduced, and the counter is more precise due to the smaller time step.

## 3. System Model

**Figure 1:**
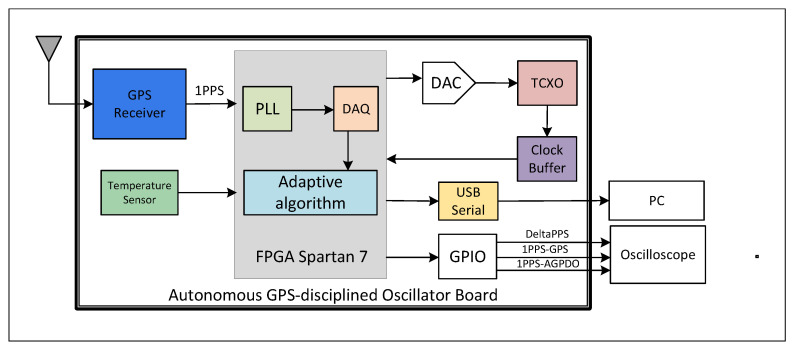
Block diagram of the autonomous GPS-disciplined oscillator.

Figure 1 illustrates the FPGA-based autonomous GPS-disciplined oscillator (AGPSDO) board and the external hardware verification. The AGPSDO board includes an FPGA Spartan 7 chip, where the phase lock loop (PLL), data acquisition module, and an adaptive algorithm are implemented, and the external peripheral devices are a 10 MHz TCXO, the universal serial bus (USB) to universal asynchronous receiver–transmitter (USB-UART) converter, GPS receiver module, and temperature sensor. A GPIO connector is used to test and observe the important signals on the oscilloscope. The USB-UART converter is deployed to collect data on the computer for the test, verification, and evaluation.

An algorithm called the adaptive oscillator model (AOM) models the frequency drift characteristics of the TCXO. This model is used when the GPS time reference signal is lost. This algorithm is composed of two Kalman filters to adaptively model the frequency drift of the TCXO due to temperature and component aging. In addition, the AOM uses three infinite impulse response filters (IIRFs). IIRF1 attenuates the GPS receiver noise. IIRF2 attenuates the temperature component of the correction signal, but passes the aging component. Subtracting the output of IIRF2 (aging) from the output of IIRF1 gives the temperature component. This temperature component is fed into the IIRF3 to further attenuate the GPS receiver noise and glitches due to the subtraction. A training controller can choose between three modes of operation, depending on the status of the GPS satellite lock: the normal mode on which the AOM is trained; the average mode if the AOM has not had enough time to train, i.e., 2 h; the hold-over mode provides the correction predicted by the AOM, which loops the output of the Kalman filters back to their input.

### 3.1. Kalman Filter Design

The state estimation equation of the Kalman aging model is defined as follows.

(1) $$x_{k+1}=\phi_{k}\times x_{k}=\left[\begin{array}{ccc} 1 & \Delta t & 0\\ 0 & 1 & \alpha \times \Delta t\\ 0 & 0 & 1 \end{array}\right]\times \left[\begin{array}{c} \frac{\Delta f}{f_{0}}\\ \frac{d}{dt}\left(\frac{\Delta f}{f_{0}}\right)\\ \frac{d^{2}}{dt^{2}}\left(\frac{\Delta f}{f_{0}}\right) \end{array}\right].$$

In the state transition matrix $\phi_{k}$, Δt is the time step. Parameter α is set when the GPS signal is available and is 0 when the system switches to hold-over, in order to have a purely linear prediction model. The state vector is denoted as $x_{k}$, while $\frac{\Delta f}{f_{0}}$ is the TCXO frequency stability. $\frac{d}{dt}(\frac{\Delta f}{f_{0}})$ and $\frac{d^{2}}{dt^{2}}\left(\frac{\Delta f}{f_{0}}\right)$ are the first and second derivative of the stability with respect to time, respectively.

The state estimation equation of the Kalman temperature model is calculated as follows.

(2) $$y_{k+1}=\phi_{k}\times y_{k}=\left[\begin{array}{ccc} 1 & \Delta T & 0\\ 0 & 1 & \beta \times \Delta T\\ 0 & 0 & 1 \end{array}\right]\times \left[\begin{array}{c} \frac{\Delta f}{f_{0}}\\ \frac{d}{dt}\left(\frac{\Delta f}{f_{0}}\right)\\ \frac{d^{2}}{dt^{2}}\left(\frac{\Delta f}{f_{0}}\right) \end{array}\right].$$

ΔT is the temperature step, and the derivatives concern the temperature. The temperature step is calculated from the measurements from the temperature sensor. β is the same constant as α.

### 3.2. Adaptive Algorithm

**Figure 2:**
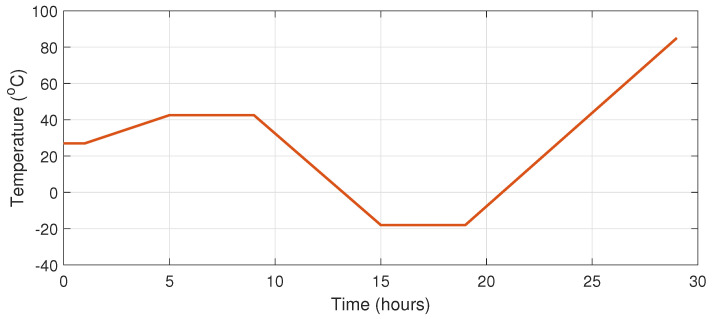
Temperature variation model.

To verify the conceptual operation of the Kalman filters, we simulated the frequency variation from the stability of the TCXO that we used on the board. The maximum values of frequency variation caused by temperature and aging were chosen. The variation due to temperature was 3.5 ppb C, and the variation was due to aging of 1 ppb/24 h. The temperature profile applied by the simulation to the TCXO was created by a temperature chamber, as shown in Figure 2.

**Figure 3:**
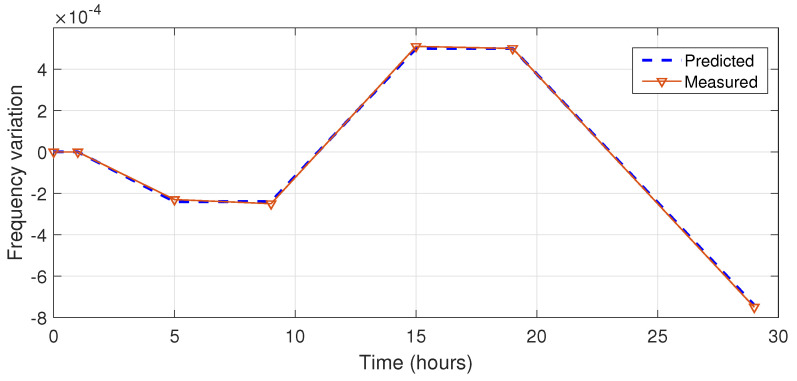
Predicted and measured total frequency variation.

Then, the aging component is fed into the input of the Kalman aging model. The temperature component is used as the input of the Kalman temperature model. The total variation includes both aging and temperature frequency variation. The result of the simulation of the system that switches to hold-over after 2 h is shown in Figure 3.

## 4. Hardware Implementation

**Figure 4:**
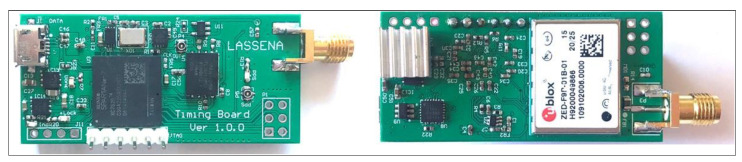
Hardware design for the proposed autonomous GPS-disciplined oscillator (AGPSDO).

The hardware implementation comprises two parts: an autonomous GPS-discipline oscillator and an external hardware verification, as shown in Figure 4. The device has a very compact size of 40×27 mm.

### 4.1. Autonomous GPS-Disciplined Oscillator

The autonomous GPS-disciplined oscillator board provides a high-stability clock source for timing applications. The GPS clock frequency is constantly monitored by the Spartan-7 FPGA chip:

- FPGA chip: The Spartan 7 XC7S25-1CSGA225C device in 225-pin BGA, which includes 3650 slices containing 4 6-input LUTs and 8 flip-flops each, was chosen for its small size and being powerful enough for software development. An internal clock frequency of 450 MHz was used to generate a 200 MHz clock for the counter.
- GNSS module: In this project, we used the ZED-F9P module, which provides multi-band GNSS to high-volume industrial applications in a compact form factor. ZED-F9P is a multi-band GNSS module with an integrated u-blox multi-band RTK technology for centimeter-level accuracy. The module enables precise navigation and automation of moving industrial machinery by means of a small, surface-mounted module. The ZED-F9P GNSS module has a serial data communication interface, a timing pulse connected to the FPGA, and serial communication.
- Temperature sensor: This board has an integrated temperature sensor to monitor the board temperature. The sensor collects temperature data and communicates to the FPGA chip using I2C communication.
- Voltage-controlled temperature-compensated crystal oscillator (VC-TCXO): A low-cost and low-accuracy oscillator was used to generate a 10MHz frequency signal.

**Figure 5:**
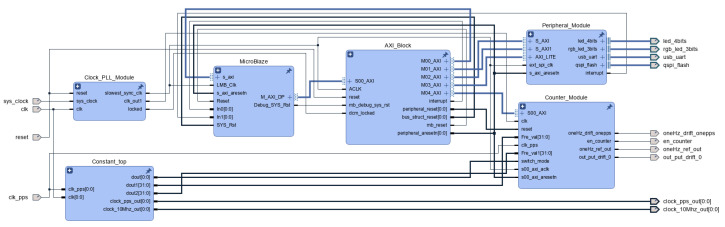
Top block diagram of the Spartan-7 chip in Vivado Design Suite.

The following part presents the hardware design inside the FPGA Spartan 7chip, which has the top block diagram as depicted in Figure 5. The microBlaze microprocessor, which provides standard connectivities to the peripheral devices such as the USB-UART device and GPIOs using the advanced extensible interface (AXI) connection was developed and integrated in the FPGA chip.

#### 4.1.1. MicroBlaze

The MicroBlaze processor, which provides multiple communication protocols such as UART, SPI, $I_{2}C$, and various general purpose inputs–outputs (GPIOs) was used. The adaptive algorithm was implemented in this processor using the C/C++ programming language. The output data from the MicroBlaze microprocessor were collected and observed in real-time on a computer using MATLAB Simulink. A low clock frequency at 50 MHz was used for the processor to reduce power consumption. The block diagram of the processing system has 3 main parts: MicroBlaze controller, AXI interrupt controller, and MicroBlaze local memory. The Microblaze controller part is the main component, which has a microcontroller configuration mode to calculate the floating-point number in the adaptive algorithm. The AXI interrupt controller manages all interrupts in our firmware: UART, timer, $I_{2}C$, and quad serial peripheral interface (QSPI). The MicroBlaze memory is a virtual RAM inside the FPGA built by the look-up tables (LUTs) of the FPGA. In this design, its capacity is 16 kB, in total, and the capacity can be expanded depending on the complex algorithm on MicroBlaze.

#### 4.1.2. AXI Connection

The AXI-LITE module was used to create the interfaces of other intellectual property (IP) modules with MicroBlaze. This module provides the parallel interfaces, and the data from other modules are transferred to MicroBlaze immediately and otherwise. This allows the C source code on MicroBlaze to process the data in real-time. This is an advantage of the firmware on the FPGA compared to other solutions.

#### 4.1.3. Peripheral Module

The peripheral module includes a QSPI module, a $I_{2}C$ module, and a UART module. The QSPI module was used to provide the interface to the Spartan 7 FPGA and the Flash chip to store the bitstreams of the FPGA. First, the Flash chip is detected via the SPI interface by the JTAG programmer. Then, the interrupts are used to receive and send data to and from the Flash.

The $I_{2}C$ module was used to interface the FPGA and the temperature sensor chip by the $I_{2}C$ interface standard. The temperature sensor collects the temperature values of the oscillator on the board, which is used in the adaptive algorithm on Microblaze to compensate and adjust the control parameter to the 1PPS module.

The UART module was used to provide the communication between the application of the FPGA and the external devices via the UART to USB chip. This module was used to display debug information.

#### 4.1.4. Counter Module/Data Acquisition Module

**Figure 6:**
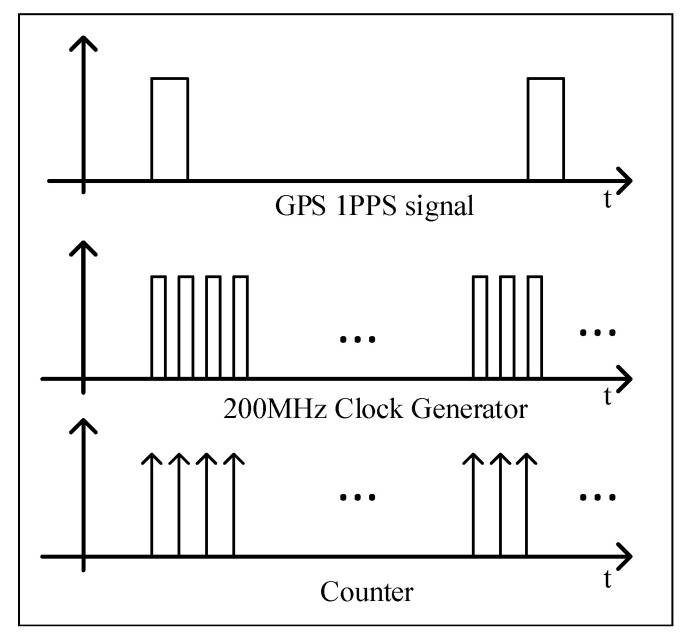
GPS-disciplined signal sampling at DAQ.

The 1-pulse-per-second (1PPS) signal received from the GPS was used to discipline the number of pulses from a high-frequency clock generated by the FPGA. Figure 6 illustrates the sampling clock (high frequency at 200 MHz) disciplined to 1PPS. A rising-edge counter was used to count the number of samples within a one-period clock cycle of 1PPS. That is, 1PPS provides the phase reference for the counter block, which produces the sampling clock [22,23]. The sampling time is guaranteed by the universal availability of 1PPS signals at different geographical locations. The number of counters is determined by the intrinsic accuracy of the 1PPS signal, the quality of the receiver, and the quality of clock sampling. A 200 MHz clock frequency was used and generated by the FPGA, which provides a high-accuracy clock generator compared to previous designs, which used a low frequency. A sampling rate of 200 MHz was used, yielding nominally 200 M samples per cycle. The counter number was transferred to the main processor of MicroBlaze by the AXI-LITE module.

### 4.2. External Hardware Devices

To collect the experimental data and verify the working conditions, the external hardware components included a computer and an oscilloscope. The following part presents the external tools and software we used to verify the proposed AGPSDO:

- Computer: The AGPSDO device also can be connected directly to a computer. The data were collected from the AGPSDO device and transferred to the computer using the UART protocol. On the computer, MATLAB Simulink was used for signal processing and displaying and plotting the data. The temperature and ΔPPS values were collected in real-time.
- Oscilloscope: The digital storage oscilloscope is a useful device to observe analog and digital signals. Moreover, the difference between 1PPS signals was captured and stored.

## 5. Measuring Oscillator Frequency

There are mainly three methods for time and frequency measurements: time interval measurement, frequency measurement, and period measurement. Time interval measurement is mostly for sine wave or PPS signals, and will not be further detailed. In the frequency measurement method, the number of cycles of the oscillator clock is measured during a certain period of time. In the period measurement method, the number of cycles of other higher-frequency clocks is measured during an integer number of cycles of the oscillator clock. In this work, we used the frequency measurement method for measuring the period error and the normalized frequency error.

**Figure 7:**
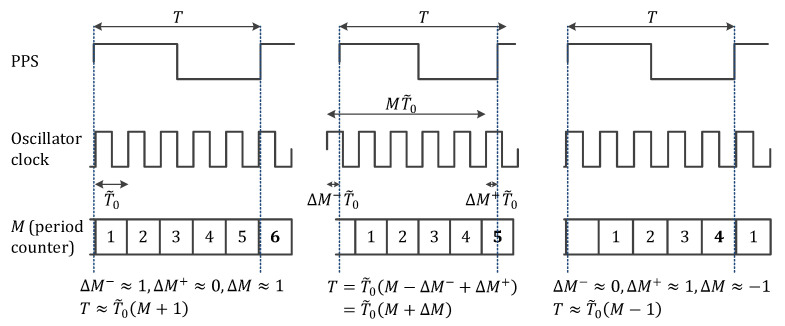
Illustration of the frequency measurement method. *T* is fixed between the three cases; the oscillator clock is slightly different. Left: worst case with one cycle counted too much; middle: average case, right: worst case with one cycle not counted.

In the frequency measurement method, illustrated in Figure 7, the actual period and frequency of the oscillator clock can be expressed as:

(3) $$T=\left(M+\Delta M\right)\tilde{T_{0}}\to \tilde{T_{0}}=\frac{T}{\left(M+\Delta M\right)}\to \tilde{f_{0}}=\frac{\left(M+\Delta M\right)}{T},$$

with *M* an integer and ΔM a real number between −1 and 1. To be more accurate, $\Delta M=\Delta M^{+}-\Delta M^{-}$, in which $\Delta M^{+}$ is the portion not counted after the last rising edge and $\Delta M^{-}$ is the portion counted too much before the first rising edge. Both of them are random variables with a uniform distribution between 0 and 1. Since ΔM is the difference of these two random variables, it is a random variable with a triangular distribution between −1 and 1. However, during the measurement, only an integer number of cycles can be measured; thus, the part ΔM is not measured by the counter, and the estimate is within ±1 clock cycle of the reality. Therefore, the measured (or estimated) period and frequency of the oscillator clock are obtained as:

(4) $$T=M\hat{T_{0}}\to \hat{T_{0}}=\frac{T}{M}\to \hat{f_{0}}=\frac{M}{T}.$$

The period error and normalized period error are thus:

(5) $$\begin{array}{c} \Delta \hat{T_{0}}=\hat{T_{0}}-\tilde{T_{0}}=\frac{\Delta MT}{M(M+\Delta M)}=\frac{\Delta M}{M}\tilde{T_{0}},\\ \Delta \hat{T_{0}^{\prime }}=\frac{\hat{T_{0}}-\tilde{T_{0}}}{\tilde{T_{0}}}=\frac{\Delta M}{M}=\frac{\Delta M}{\tilde{f_{0}}T-\Delta M^{\prime }}. \end{array}$$

The frequency error and normalized frequency error are calculated as:

(6) $$\begin{array}{c} \Delta \hat{f_{0}}=\hat{f_{0}}-\tilde{f_{0}}=\frac{\Delta M}{T},\\ \Delta \hat{T_{0}^{\prime }}=\frac{\hat{T_{0}}-\tilde{T_{0}}}{\tilde{T_{0}}}=\frac{\Delta M}{M}=\frac{\Delta M}{\tilde{f_{0}}T-\Delta M^{\prime }}. \end{array}$$

Note that the worst case ΔM=±1 can happen during one measurement, but not on several consecutive measurements. Indeed, looking at Figure 7, for the measurement period shown, there is a clock rising edge just after the PPS rising edge, whereas for the next measurement period, there is a clock rising edge just before the PPS rising edge; therefore, the “configuration” is different, and it is not possible to have the worst case on consecutive measurement periods. This is illustrated in Figure 7. This means that the errors made in consecutive estimates are not independent; M+ΔM is always the same since *T* and $\tilde{T_{0}}$ are assumed fixed, and since *M* can change by ±1, the current ΔM is the previous one ±1; consequently, consecutive ΔM are not independent. This point will be important later for counting the time because the time counted relies on consecutive measurements.

Note that it is also possible to send the oscillator clock to a phase-locked loop (PLL) that will multiply the frequency, and it is the cycles of this high-frequency clock that will be counted. In this case, if the multiplication factor of the frequency is *P*, the periods and frequencies become:

(7) $$\begin{array}{c} T=\left(M+\Delta M\right)\frac{\tilde{T_{0}}}{P}\to \tilde{T_{0}}=\frac{PT}{M+\Delta M}\to \tilde{f_{0}}=\frac{M+\Delta M}{PT},\\ T=M\frac{\hat{T_{0}}}{P}\to \hat{T_{0}}=\frac{PT}{M}\to \hat{f_{0}}=\frac{M}{PT}. \end{array}$$

Then, the error and normalized error become:

(8) $$\begin{array}{c} \Delta \hat{T_{0}}=\frac{\Delta MPT}{M(M+\Delta M)}=\frac{\Delta M}{M}\tilde{T_{0}},\Delta \hat{T_{0}^{\prime }}=\frac{\Delta M}{M}=\frac{\Delta M}{Pf_{0}T},\\ \Delta \tilde{f_{0}}=-\frac{\Delta M}{PT},\Delta \tilde{f_{0}^{\prime }}=-\frac{\Delta M}{P\tilde{f_{0}}T}=-\frac{\Delta M}{M+\Delta M^{\prime }}. \end{array}$$

From these equations, it is shown that the higher the measured frequency, the lower the error.

## 6. Experimental Results

### 6.1. Clock Synchronization

**Figure 8:**
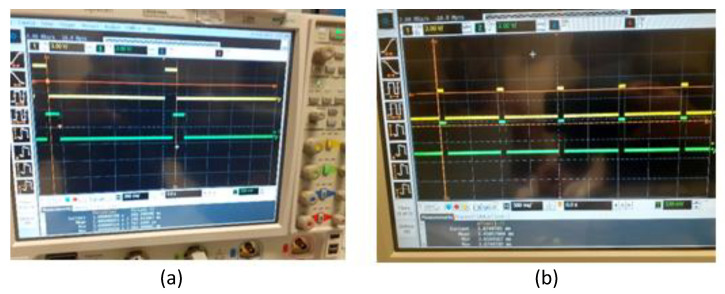
(**a**) Imperfect alignment before modification in the FPGA firmware. (**b**) Perfect alignment of 1PPS.

Due to computation time in the data acquisition phase and software-adaptive algorithm in the FPGA chip, there was a constant delay between the 1PPS signal from the GNSS receiver module and the generated 1PPS signal, which was measured as 145 ms. The sawtooth correction method and the counter clock adjustment techniques are some of the clock synchronization techniques used to synchronize the timing signal employed in the literature [3,24]. The phase alignment between the GNSS and atomic clock is essential for an unbiased and deterministic time distribution downstream. A typical GNSS receiver provides the time reference through its output 1PPS signals. The output 10MHz frequency signal from GNSS follows the 1PPS coherently. The derivative of the 1PPS phase in time is consistent with the output frequency. The unknown phase relationship between the PPS and 10 MHz signals from GNSS becomes irrelevant; this offset may be a constant one, and it is strictly bounded within ±1(ns). Sometimes a time difference may arise due to the change in the electronically advanced or delayed internal clock signal to generate the 1PPS signal aligned to GPS time. The adjustment of the time difference between the two 1PPS signals is carried out in the firmware of the FPGA. The perfect alignment after modifying the FPGA firmware is illustrated in Figure 8.

### 6.2. Temperature Dependency of Frequency

**Figure 9:**
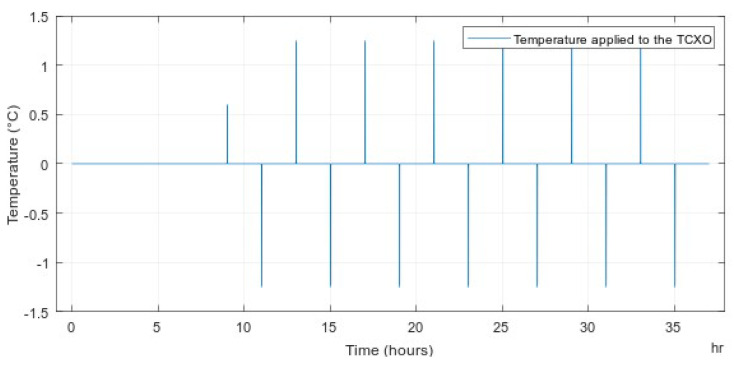
Temperature variation applied to the TCXO on the AGPSDO board.

**Figure 10:**
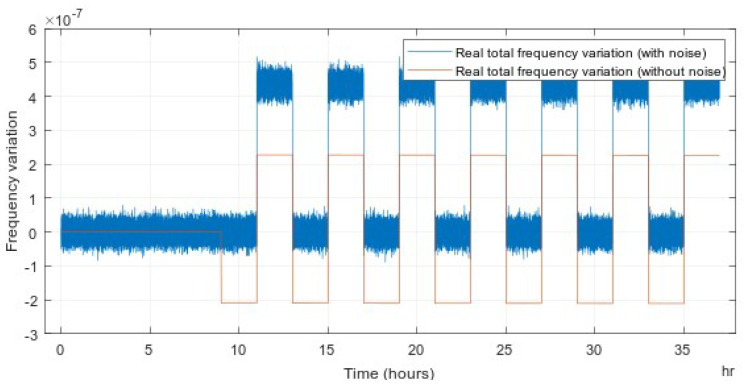
Frequency variation due to the change of temperature on the board.

To evaluate the effect of temperature on the board’s performance, the frequency varies according to the change of temperature as observed from the TCXO on the AGPSDO board. The board was set up in a stable temperature chamber. The temperature was controlled to vary in the range of $\pm 1.2{\;}^{\circ }$C. The temperature profile applied to the TCXO is shown in Figure 9. The temperature variation was the input of the adaptive Kalman temperature model. The board was switched to hold-over mode after 2 h of learning mode. The result of frequency variation with noise and with the noise filter is described in Figure 10. The temperature variation contributes significantly to the frequency of TCXO at the maximum of 3.5 ppb/${}^{\circ }C$. Therefore, the temperature compensation is necessary to achieve a high-precision timing clock.

### 6.3. Measurement Results

In the measurements, we use the term ΔPPS (DeltaPPS), which is the timing error between two adjacent PPS pulses [19]. The 1PPS GPS received signal and the 1PPS signal from the AGPSDO have a relationship:

(9) $$1s=\left(t_{n}+t_{offset_{n}}\right)-\left(t_{n-1}\right)-t_{offset_{n-1}}).$$

The PPS time intervals were measured during the test; therefore, the difference between two adjacent 1PPS signal is the measured ΔPPS, which is expressed as:

(10) $$\Delta PPS=t_{offset_{n}}-t_{offset_{n-1}}=1s-\left(t_{n}-t_{n-1}\right).$$

#### 6.3.1. Indoor Experimental Result

**Figure 11:**
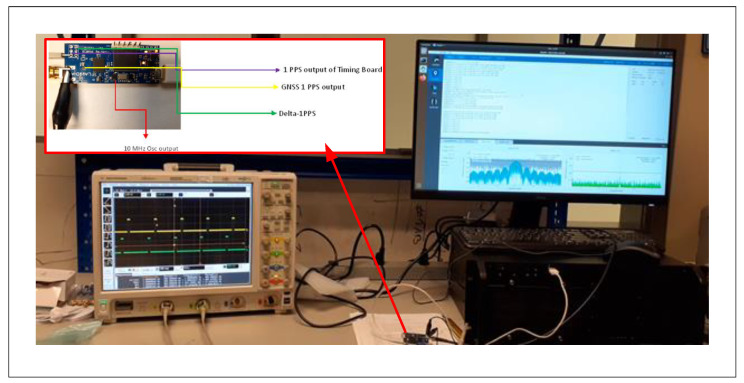
Experimental setup for indoor measurement.

The experimental setup for the indoor test is depicted in Figure 11. The setup included a digital storage oscilloscope (DSO) to capture and observe the real-time signals including the GNSS 1PPS signal and the 1PPS signal generated from the AGPSDO board. A computer was used to collect signals from UART ports (GNSS 1PPS signal and generated 1PPS signal). Based on the setup shown in this figure, the advanced GNSS simulator Orolia was connected to the AGPSDO board, and the Dektec DTA-2115B SDR card generates the GPS $L_{1}$ signal at a sampling rate of 12.5M−samples/s coming from the four RF channels combined to connect the DC-block to the antenna port of the GNSS receiver. The power level of the simulated signal was set at −130 dBm, and the default gain of 50 dB was chosen during the lock-in mode of the receiver. The second channel of the DSO was allocated to know the readings, and in the Agilent scope, the settings of the measurement mode of difference between the channels in the two rising edges were kept for finding the deviation between the signals.

**Figure 12:**
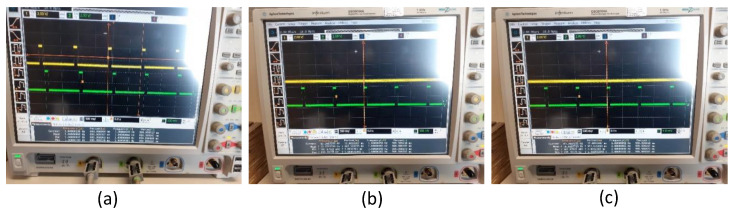
DSO measurements: (**a**) 1PPS measurement, GNSS is ON; (**b**) 1PPS measurement, GNSS is OFF at the beginning; (**c**) 1PPS measurement, GNSS is OFF after 24 h.

At the beginning of the initial measurement, the lock-in mode that is the GNSS signal was ON; in this mode, the difference between the two signals is noted, and at the start of the first hour, the interference option was set up in the GNSS simulator so that after a fraction of seconds, slowly, the availability of the GNSS signal was vanishing. Then, the time of the 1PPS timing signal was examined after 24 h (1 day), and the behavior of the timing signal was carefully verified to see the stability of the signal generation in the FPGA firmware concerning the amplitude and the time duration as in Table 1. We found that the readings were good enough to achieve the anticipated variations in the UART console of the FPGA, as well as in the real-time reading from the DSO, as depicted in Figure 12.

**Figure 13:**
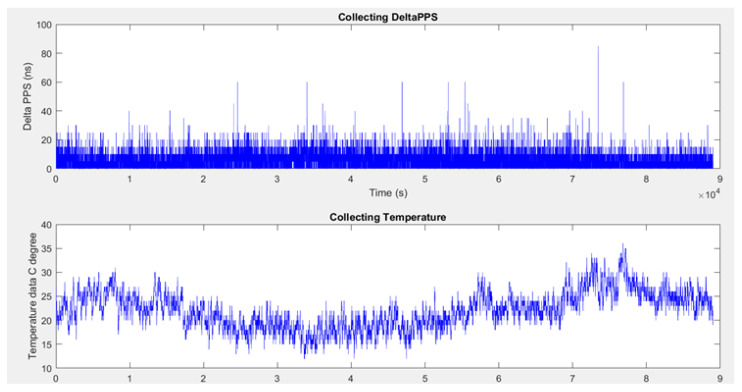
Measurement results of the ΔPPS and temperature indoors.

**Figure 14:**
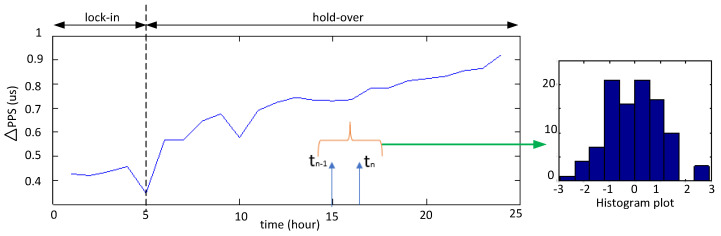
Real-time ΔPPS tested with the Orolia GNSS simulator and its distribution.

Figure 13 shows the collected ΔPPS and the temperature measurement for the indoor experiment. The histogram plot presented in Figure 14 for a difference between the two integer time instants is given, and the respective changes in the histogram plot were also observed in this period. To evaluate the long-term stability of the PPS, we kept it fluctuating around the GPS integer seconds, and it had about a zero cumulative error. In other words, the PPS offsets from the corresponding GPS integer seconds can be regarded as a zero-mean Gaussian variable. The assumptions considered that the offsets of the difference between two time instants after the hold-over period obey a Gaussian distribution. Therefore, the difference between two adjacent offsets $t_{offset_{n}}-t_{offset_{n-1}}$ obeys the Gaussian distribution.

#### 6.3.2. Outdoor Experimental Result

**Figure 15:**
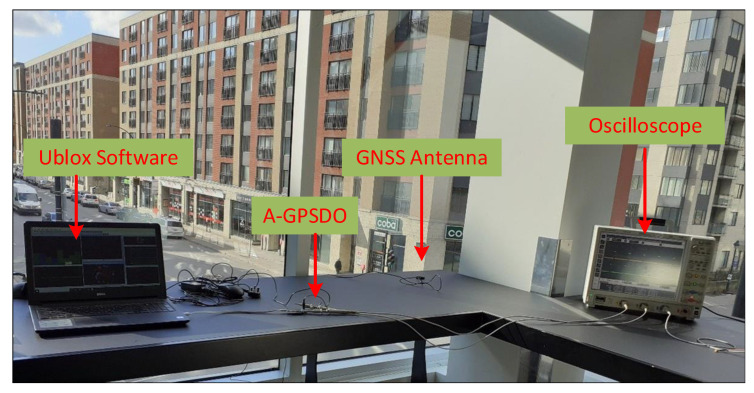
Experimental setup for the outdoor environment in the corridor.

To evaluate the real-time measurements that were clock accurate (atomic clock) from the satellites based on live signals, it was decided to record and playback the live sky signals rather than the generated GNSS signals. First, the test in the corridor of the first floor was set up as depicted in Figure 15. The GNSS antenna was connected to the RF input port of the AGPSDO board. For blocking the satellite signals, we used the blade RF to generate a single-tone CWI to disable the detection of the GNSS signal on the AGPSDO device. The interference setup in Simulink of the RFI generation was used. On the other hand, the satellite visibility was observed from the UCenter software installed on a laptop. The lock-in period was kept within only one hour since the continuous reception of the GNSS signal is a challenging task in this indoor arena, so we restricted it only to 1 h. In this lock-in period, the initial reading of 1PPS of both pulse signals was recorded, then we enabled the blocking of satellite signals at the start of the second hour; both signals were observed to see the difference in ΔPPS. Then, we continuously measured the readings of the hold-over period over a day.

The measurements were not stable because the receiver switched from lock-in mode to hold-over mode rapidly due to the low signal power; thus, one can notice much variation in the mean value of the 1PPS signal for a 24 h duration. The values obtained in this experiment may not be accurate to evaluate the difference. For example, for the difference between the reference signal and the timing signal from 2 h to 2 h 5 min, (5 min of observation time), the switching occurred 11 times; therefore, the calculation of ΔPPS, which is relevant to the reference GPS signal, is a difficult task to measure and to evaluate the changes in ΔPPS.

From the tabulated results in Table 2, the mean difference of 1 PPS for the first-hour lock-in mode showed 0.00378 μs after changing the mode of hold-over and a shift of 0.225 → 0.767 μs in the 1PPS measurements for the first 5 min. At the end of one hour, the variations kept under the required level of 1/10 μs. However, the drift changed to 0.178 μs, slightly beyond the threshold value, after 2 h.

### 6.4. Comparison to the Reference Boards

**Figure 16:**
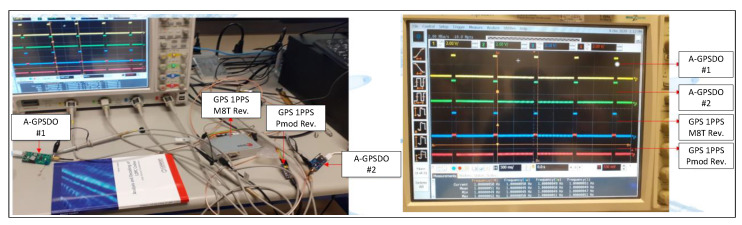
Measurement setup with different reference boards and the results.

In this part, we provide measurement results of the generated 1PPS signal with several reference boards. Most of the measurement procedures of comparing the timing signal based on the reference receivers have been given in several references [25]. To distinguish between two timing signals in hold-over mode, we needed a reference signal to see the time difference after lock-in mode, so we decided to employ four GNSS receivers to confirm that the pattern of the PPS signal was the same for all cases. In our experiment, the Ublox GPS M8T receiver, the Pmod GPS, and the two-timing boards having the same Ublox ZED-F9P receivers were used to see the functioning and the deviation of the 1PPS GNSS signal relevant for all 1PPS signals of the reference receivers. As illustrated in Figure 16, all the receivers were connected to the GNSS simulator with the same GPS $L_{1}$ signals simultaneously, and their corresponding 1PPS signals were displayed in the four channels of the DSO, respectively.

### 6.5. Discussion

In this part, we provide the comparison of the proposed AGPSDO and the previous works in Table 3. The work in [11] achieved a time error of 1.5 μs over a 24 h hold-over period, then the frequency stability was 0.017 ppb. The authors also presented an adaptive algorithm to compensate the temperature effect, and the achievement met the timing requirement for the CDMA network base station. However, the hardware design and the power consumption were not provided. The authors in [16] provided the hardware implementation of the GPSDO, which aimed to reduce the power consumption of the wireless sensor nodes. The above-mentioned GPSDO achieved a 5.03 μs time drift within a 1800 s window time of disciplining, which consumed below 100 mW. However, the frequency stability was low considering the hold-over time of 1800 s in the experiment. Our proposed AGPSDO achieved a 0.004 ppb frequency stability while consuming 600 mW. It is noted that commercial products that provides higher frequency stability (1 ppb) consume around 500 mW.

## 7. Conclusions

In this paper, we presented our proposed autonomous GPS-disciplined oscillator in an FPGA-based hardware design, which was aimed for use in wireless sensor network nodes. The timing requirement for the sensor nodes could be achieved by using the proposed AGPSDO. The design, which takes advantage of the high frequency in data acquisitions and an adaptive algorithm for calibration, provided high accuracy for the timing reference board. Experimental results with indoor and outdoor environments verified the accuracy of the proposed algorithm and hardware implementation of the autonomous GPS-disciplined oscillator. The AGPSDO board showed that it had only an 80 ns frequency drift after 24 h of working without GPS signals. The frequency stability achieved 0.004 ppb with the outdoor experiment, proving the advantages of the proposed hardware implementation and software design. The timing performance was compared to the commercial reference boards and the state-of-the-art. In the future, sensor nodes using the proposed AGPSDO could be implemented and tested in real-time applications.

## Figures and Tables

**Table 1:** Measurement of ΔPPS in an indoor environment with hold-over mode.

Measurements	GPS ON	GPS OFF	GPS OFF	GPS OFF	GPS OFF	GPS OFF	GPS OFF
		Hold-Over	Hold-Over	Hold-Over	Hold-Over	Hold-Over	Hold-Over
	Mean Time	Min. Time	Max. Time	Mean Time	Min. Time	Max. Time	Mean Time
	(Start of 1 h)	(End of 1 h)	(End of 1 h)	(End of 1 h)	(End of 24 h)	(End of 24 h)	(End of 24 h)
1PPS GPS (ms)	999.999397	—	—	—	—	—	—
1PPS AGPSDO (ms)	999.999341	999.998973	999.998901	999.999347	999.999419	999.999597	999.999609
Δ*PPS* (ms)		0.000020	0.000042	0.000050	0.000072	0.000068	0.000080
		(0.002 μs)	(0.042 μs)	(0.005 μs)	(0.072 μs)	(0.068 μs)	(0.080 μs)

**Table 2:** Measurement of ΔPPS in the outdoor environment with hold-over mode.

Measurements	GPS ON	GPS OFF	GPS OFF	GPS OFF	GPS OFF	GPS OFF	GPS OFF
		Hold-Over	Hold-Over	Hold-Over	Hold-Over	Hold-Over	Hold-Over
	Mean Time	Min. Time	Max. Time	Mean Time	Min. Time	Max. Time	Mean Time
	(Start of 1 h)	(End of 1 h)	(End of 1 h)	(End of 1 h)	(End of 24 h)	(End of 24 h)	(End of 24 h)
1PPS GPS (ms)	999.999397	—	—	—	—	—	—
1PPS AGPSDO (ms)	999.999341	999.998973	999.998901	999.999347	999.999419	999.999597	999.999609
Δ 1PPS (ms)		0.000020	0.000042	0.000050	0.000072	0.000068	0.000080
		(0.002 μs)	(0.042 μs)	(0.005 μs)	(0.072 μs)	(0.068 μs)	(0.080 μs)

**Table 3:** Comparison of the proposed AGPSDO and the previous works.

GPSDO	Hold-Over Time	Δ *PPS*	Frequency Stability	Power Consumption
[11]	24 h	1.5 μs	0.017 ppb	–
[16]	1800 s	5.03 μs	–	100 mW
This work	24 h	0.356 μs	0.004 ppb	600 mW

## Data Availability

Not applicable.
